# Integrated Health and Social Care in England: Ten Years On

**DOI:** 10.5334/ijic.5666

**Published:** 2021-10-29

**Authors:** Robin Miller, Jon Glasby, Helen Dickinson

**Affiliations:** 1 University of Birmingham, UK; 2 University of New South Wales, Australia

**Keywords:** England, policy, integrated care, partnership working, leadership

## Abstract

**Introduction:**

As part of major policy reforms begun in 2010, England introduced a wave of initiatives to encourage more integrated care between health and social care. These built on previous attempts which sought to achieve similar objectives through a focus on better partnership working. This article provides an overview and critical commentary on integrated care policy in England from 2010–2020 based on reviews by regulators, parliamentary committees and the national audit office.

**Overview of Policy:**

Integrated care became a priority through the work of the Future Forum, a group of leading stakeholders established due to concerns about greater competition in public health care. This led to a public statement of shared commitment to integrated care by national health and social care bodies. Early mechanisms included a pooled fund to achieve nationally set objectives, the creation of local authority led partnership boards, and high profile innovation programmes. Later in the 2010’s, new health led partnerships became more dominant vehicles to achieve integrated care at regional level.

**Impact of Policy:**

Despite progress within a few local areas, and reduction in delayed discharges from hospital the overall picture from national reviews was that expected improvements were not achieved. Emergency admissions to hospital continued to grow, patients within primary care reported being less involved in their care, and health inequalities worsened. The initial response to COVID-19 was health-centric contributing to outbreaks in care homes and inadequate supplies of protective personal equipment. The ability of leaders to look beyond their organisations’ interests was reported as vital for local progress. National government performance frameworks discouraged system based working and chronic underfunding of social care led to major capacity and workforce challenges.

**Conclusion:**

The experience of England suggests that greatest progress is made when integrated care focusses on tangible issues and when there is a clear understanding of how success will be measured. Even with considerable investment and intent progress should be expected to be slow and difficult. Layering of numerous policy initiatives provides confusion and can distract from the important work of relationship building. And ultimately, integrated care cannot by itself address major inadequacies in the underlying resources and structural inequalities.

## Introduction

A decade ago we published a paper within this journal [1] exploring partnership working between English health and social care organisations, summarising the key approaches adopted and whether this had resulted in positive impacts [either for those delivering care or for people receiving it]. In this paper we explore the experience of the past decade following this initial paper to explore whether progress has been made and what this tells us about collaborative working in health and social care settings. As such, this paper provides insights for an audience interested in the specific English context, but also generates lessons about how to drive joined-up working and the pitfalls to avoid that are relevant for broader international learning of integrated care. It also starts to explore how key concepts and policy agendas may evolve over time in response to significant national and international challenges such as the financial crisis of 2008 or COVID-19 and how much continuity there can be, even with governments of very different political persuasions in office. To understand policy aspirations and the progress achieved, we draw on reports by relevant governmental bodies. This includes reviews by the regulator of health and social care (Care Quality Commission), the independent spending watchdog (National Audit Office), and scrutiny committees within parliament (Committee of Public Accounts and Health and Social Care Committee). These bodies draw on a range of sources including directly gathering the views of people with lived experience of health and care services and/or engaging with their representative bodies. The authors are academics who have undertaken independent research of integrated care policy reforms in England over two decades.

## Partnership working in health and social care

In exploring whether we have seen significant progress over the past decade we first need to revisit the context as it was in 2010. Health care in England has traditionally been coordinated and delivered through local planning and provider organisations which are part of the NHS. Whilst local NHS bodies have a degree of autonomy they have ultimately been accountable to the national government. Social care has been the responsibility of local authorities which have their own democratically elected governance structures. Local authorities follow national policy but have more autonomy than the NHS to decide on how these are implemented and how available funding is deployed. As we outlined at that time, the need for joint working across health and other agencies had been recognised for as long as the UK welfare state had been around. From the mid-1970s until the late 1990s there had been a range of consultative and planning committees established to encourage joint working in addition to a statutory duty for health and local government to collaborate. From the late 1990s, however, we saw a step change in the policy arena as partnerships emerged as a key priority area. A range of different organisational forms such as Care Trusts [2] and Children’s Trusts [3] were initiated alongside various changes to legal powers and policy exhortations [4]. These organisations were responsible for the planning and purchasing of services [i.e. commissioning in English terminology] and/or the delivery of community services. Over this period, the case was firmly made that collaboration was a necessity between health and social care. Indeed, this agenda was so firmly pressed that arguably it became heretical to even challenge the idea that partnerships were needed [5]. As we stated in 2011, ‘it has become increasingly clear that people do not live their lives according to the categories created in our welfare systems – and some form of joint working is essential if we are to find meaningful ways of joining up services in order to meet complex needs more fully’ [1: pg. 7].

Between 1997 to 2010, New Labour exhorted, compelled and incentivised agencies to work in partnership through a variety of means. For the most part, the governments of this period were not prescriptive about the form health and social care partnerships should take and seemingly allowed a level of local discretion in terms of how agencies, organisations and individuals would work together. National government focused on creating a context that was receptive to partnership working, promoting a series of different organisational structures that local areas could explore if they felt that they were right for them, and removing legislative boundaries to closer joint working. Yet, this focus on the macro level may have come at the detriment of the local level, where the actual work of collaboration is done [6]. As we argued in 2011, successive English governments had been unduly focused on the ‘structural’ elements of partnership working, which failed to acknowledge the reality of the ways that organisations and professionals develop relationships and trust in order to be able to work together. Joint working is about more than simply the removal of organisational and legislative boundaries, and the continual reorganisation of health and social care agencies continued to hamper attempts of local organisations in working together. It is perhaps unsurprising that a number of different studies remained skeptical as to whether partnerships had proved able to deliver significant outcomes [7,8,9,10]. However, as a caveat to this statement, a number of evaluators noted that the research evidence is not replete with rigorous studies of the outcomes of partnership working and that the challenges in undertaking this kind of research are plentiful [11,12].

## What happened next?

As summarised above and in our original article [1], between 1997 and 2010 successive Labour governments placed significant emphasis on partnership working through a series of policy reforms. This period also saw substantial increases in health and social care funding over a sustained period, albeit the worldwide economic crisis of 2007–08 made future increases difficult for any government. In 2010, Labour lost the general election and a new government was formed by a Conservative-Liberal Democrat Coalition (2010–15). Within this government the Conservatives were particularly committed to a policy of austerity that resulted in reductions in public service expenditure. In 2015, the Coalition was replaced by a standalone Conservative government, which remains in charge at the time of writing (albeit with a series of different leaders over time). Below we set out the policy context with respect to the key issues relating to joined-up working in terms of three phases (2010–15, 2015–20 and the response to COVID-19). This is a necessarily brief account, but more detail can be found in [13,14,15].

### Integrated care under the Coalition (2010–15)

Initially, there was little mention of joint working under the Coalition government. Their reforms were focused on increasing market forces in the NHS and putting oversight of local markets in the hands of general practitioners through Clinical Commissioning Groups. However, this became a specific policy priority after being taken up by the NHS Future Forum [a group of health and social care leaders and other stakeholders set up to review the government’s 2012 health reforms, established after a significant backlash to a perceived focus on competition over collaboration]. Whereas the previous government had used terms such as ‘partnership working’ and ‘joined-up solutions to joined-up problems’, the Future Forum and the Coalition tended to talk about ‘integrated care’ (sometimes referring to vertical integration between hospitals and community, and sometimes seeming to refer to horizontal integration between health and social services). This culminated in the publication of a ‘shared commitment’ to integrated care by all of the key national governmental and independent bodies for health and social care [16]. This was focused around the definition of person centred and coordinated care that was developed by the charity National Voices for this new National Collaboration: *“I can plan my care with people who work together to understand me and my carer[s], allowing me control, and bringing together services to achieve the outcomes important to me.”* [17: p. 3].

While the notion of ‘integrated care’ was widely promoted, there felt a potential disconnect between the senior health and social care stakeholders who had genuinely championed it, and a government looking for new language and approaches to salvage its controversial health reforms. As part of these changes, public health responsibility [and associated staffing and funding] for improving the health of the local population passed from the NHS to local government. This included providing information and advice on health improvement, supporting people to adopt healthier lifestyles and researching health inequalities. Other public health responsibilities such as protecting the public from major hazards and responding to public health emergencies passed to a new national body (Public Health England). A raft of supporting legislation and nationally led initiatives were introduced (***Table 1***) These included the Better Care Fund to invest in joint priorities [with initial flexibility being reduced over time and becoming more focused on core NHS priorities such as tackling delayed hospital discharges]. The creation of Health and Wellbeing Boards within local government to bring together different partners to develop local strategies for health improvement (that seemingly had significant responsibilities and very little power to actually bring about change). Two pilot projects (the ‘Integrated Care Pioneers’ and ‘Vanguard’ sites), set up by slightly different parts of government to test new ways of joint working, arguably created a degree of overlap and confusion. Over time, there was a growing sense these were losing their initial locally-owned priorities and became dominated by targets relating to emergency hospital admissions and timely discharges from hospital.

**Table 1:** Key policy initiatives and legislation relating in integrated health and social care in England between 2010 and 2014.


YEAR	TITLE	OVERVIEW

2012	Health & Social Care Act 2012	Established local health and wellbeing boards in each local authority area, with a duty to encourage the integrated commissioning of health and social care services. Required clinical commissioning groups to promote integration where this would improve quality or reduce inequalities.

2013	Integrated Care: Our Shared Commitment	The Department of Health and twelve national partners made a commitment for urgent and sustained action with an ambition to make joined-up and coordinated health and care the norm by 2018.

2013	Integrated Care and Support Pioneers	Twenty five local areas were selected to pilot new ways of working to improve the quality and cost-effectiveness of care for people whose needs are met from both NHS and local authority services.

2013	Better Care Fund	This national initiative required clinical commissioning groups and local authorities to pool a minimum of £3.8 billion to promote integrated working, overseen by local health and wellbeing boards

2014	Care Act 2014	Required local authorities to promote integration where this would promote wellbeing, improve quality, or prevent care needs from developing

2014	Five Year Forward View	Called for a ‘radical upgrade’ in prevention and public health; models of care which shift care from hospitals to settings closer to people’s homes. Introduced seven new models of care based around the Five Year Forward View to be piloted at 50 ‘vanguard’ sites


These various different initiatives proved unable to overcome the pressures and tensions created by austerity and by the Coalition’s reorganisation of the NHS (abolishing Strategic Health Authorities, who had a regional overview of the system, replacing more managerially-led Primary Care Trusts with more clinically-led Clinical Commissioning Groups, and creating a series of new national bodies). In effect, the impression was of a series of attempts to join back together relationships and accountability mechanisms that had been swept away, and to replace functions that turned out to be essential after they were abolished.

### Integrated care under the Conservatives (2015-)

The Conservative governments between 2015 – 2020 sought not to reverse the fundamental issues of fragmentation within the health reforms but rather to introduce a series of NHS developments to recreate some of the governance and accountability mechanisms needed to help the health and social care system work together more effectively (***Table 2***). At the regional level, this included the creation of Sustainability and Transformation Plans (STPs) across 44 different areas of England, in an attempt to bring key partners together to work on system-wide issues. These were NHS bodies which had no legal standing and for whom accountability was unclear other than being required to submit their plans for approval by national bodies. STP’s varied significantly in terms of the extent that individual partners are genuinely committed to the broader partnership, and some were accused of developing plans for potentially significant service change behind closed doors. Over time, the aspiration became to develop these into ‘Integrated Care Systems’ (ICS), a deeper collaboration to take more joint responsibility for local health care system resources and performance. Interestingly, these mechanisms lacked a statutory basis, making it difficult to know where power really resides, who to hold to account and how best to bring together a series of standalone health and social care organisations who are ultimately accountable to their own Boards or local councillors, rather than to each other. At the same time there was also been a trend towards greater economies of scale, with a number of mergers taking place between local hospital, community health and mental health providers. While policy remained based on an ongoing purchasing-provider split, the reality was that significant power lay with large acute providers, who seemed increasingly dominant within their local health economies. Within general practice, General Practitioners were encouraged to form new Primary Care Networks based on local communities of around 30,000 to 50,000 people, combining local care with the economies of scale that come from multiple practices working together.

**Table 2:** Key policy initiatives and legislation relating in integrated health and social care in England between 2015 and 2020.


YEAR	TITLE	OVERVIEW

2015	Spending Review and Autumn Statement 2015:	Introduced a commitment to integrate health and social care services across England by 2020 and required local areas to submit plans by April 2017 demonstrating how they would achieve this

2015	Sustainability and Transformation Plans	Local health bodies were required to draw up plans to improve services and finances over the five years to March 2021 around identified ‘footprints’. There was a subsequent shift in focus from the ‘Plan’ to the ‘Partnerships’

2018	Integrated care systems	Advanced forms of Sustainability and Transformation Partnerships in which the local NHS organisations are awarded greater autonomy over use of available funding and managing the quality of their health care services. National bodies only assure system level plans rather than those of individual organisations. Local areas applied for this status.

2019	NHS Long Term Plan	Committed to the development of Integrated Care Systems in every area of England by 2021.

2019	Primary Care Networks	Individual general practices can establish or join PCNs covering populations of between 30,000 to 50,000 to integrate primary care services around local communities and collaborate with other relevant agencies [including social care]


### The response to COVID-19

As the effects of the COVID-19 pandemic began to spread around the world, this has intensified the need for public services such as health and social care to work together. At the local level this led to a series of very rapid and innovative bottom-up approaches, potentially overcoming years of tensions and barriers. In one locality, for example, there was joint work to tackle rising rates of domestic violence, action to reduce rough sleeping during the national ‘lockdown’, and work with supermarkets, banks, IT providers and the voluntary sector to make sure that families in need were fed, had access to emergency finance and had internet access for educational purposes [personal communication].

However, such activity often took place underneath the policy radar, and there were significant criticisms that national policy has prioritised urgent changes to the delivery of hospital services at the possible expense of others parts of the health and social care system. While a key priority was quite rightly been to reconfigure hospital services, expand intensive care and maintain as many other health services as possible, services such as care homes or supported housing felt almost entirely neglected, struggling with access to personal protective equipment, with staffing, with funding and with devastating mortality rates [18]. Even national attempts to say thank you to public sector workers helping to get society safe seemed to priortise NHS staff, with care workers feeling marginalised and under-appreciated. None of this has been caused by the pandemic as such but rather highlighted the fragmentation and vulnerability of adult social care to an even greater extent than before.

During this period the government announced that Public Health England, only created during the Conservative health reforms of 2012, would be replaced by a new National Institute for Health Protection, amidst fears that Ministers were seeking to pass responsibilities for national failures to an arms-length body. There were also been significant debates around the respective roles of the Secretary of State for Health and Social Care and NHS England [the body created in 2012 to give the NHS a degree of independence from day-to-day government intervention in the health service].

## What difference have these policies made?

The overall objectives of these various integration strategies was articulated in a cross-departmental policy statement [16]. This reflected the widely adopted ‘triple aims’ of integrated care – “individual experience of integrated care and support that is personalized and coordinated, shift away from over-reliance on acute care towards focus on primary and community care, and population based public health, preventative and early intervention strategies” [p. 13]. Given the prioritisation of integration and the considerable financial investment connected to its implementation, there was a series of reviews by Parliamentary committees and national scrutiny bodies to determine the difference this has made. These suggested a degree of progress has been made towards the over-reliance on acute care. For example, the National Audit Office [19] reported the Better Care Fund resulted in a reduction in permanent admissions of older people to care homes, and a greater proportion of older people remained at home following discharge from hospital. The Vanguards in particular were seen to be successful in reducing growth of emergency admissions to hospital [20] and there was also a steady reduction in the number of hospital beds occupied by people who were fit to be discharged but were experiencing a ‘delayed transfer of care’ between 2017 and 2019 [21].

Overall it is clear that integration of care in England has not achieved the wide variety of different aims and objectives that have been aspired to. For example, patients report that since 2012 they are less involved in making decisions regarding their primary care services and receive less support to manage their own care [22]. National data suggests emergency admissions have continued to grow and many of these are avoidable. For example, in 2016–2017 NHS England estimated that 24% were avoidable [23]. Moreover, there has been a general slowing in the increase of life expectancy with the greatest impact in areas of high deprivation. Female life expectancy has declined in the more deprived 10 per cent of neighborhoods and regional inequalities in life expectancy have also grown. In 2018 there were 69 percent more children within homeless families in temporary accommodation in 2018 than in 2010, child poverty rates have returned in the same time period to pre-2010 levels [24]. Care for particular ‘seldom heard’ groups has also been of significant concern. For example, there have been several national policy initiatives during the decade to reduce the number and length of stay of people with a learning disability and complex needs who are detained for extended periods in assessment and treatment facilities [e.g. 25,26]. Despite these, and the connected investment in partnership infrastructure, practice developments and performance monitoring, in 2020, 2,095 people were being cared for in such facilities and over 60% were subject to stays of over 2 years [27]. The “undignified and inhumane care” that many received could have been avoided through better coordination and community based support [28: p. 3].

A major issue within all forms of integrated care is the high level of variation across England. A minority of areas do appear to have made substantial progress in relation to better joint working between health and social care (for example Frimley, Nottinghamshire and Greater Manchester). However the majority have not made such bold progress and some have achieved little improvement in their ability to better collaborate across health and social care. As a consequence, cross-bench committees of Members of Parliament have questioned the government’s ability to achieve consistent integration of care [20,29,30], as have national scrutiny bodies [e.g. 23,31]:

“There are examples across England where integrated working has been successfully applied. But it is a long way from being in place everywhere, with a range of longstanding legal, structural and cultural barriers hindering the pace and scale at which change can happen.” [20: p. 3]

“The Department’s expectations of the rate of progress of integration are over-optimistic. Local areas that have achieved more coordinated care for patients from closer working between social care and NHS organisations have been doing so for up to 20 years.” [19: p. 18]

Local factors have undoubtedly had a role in this lack of consistent improvement. While some leaders are able to work beyond their organisational interests in order to respond to the needs of local communities, there are also many examples where this is not the case. Social care and the wider voluntary sector are still often excluded from strategic discussions and the pressure of responding to COVID-19 has led to greater tension [31]. The factors that have enabled better local collaboration in England are not new – a common vision focused on the local population, joint planning and funding arrangements, a supported and resourced workforce, and shared governance and communication processes [32]. Leadership continues to be highlighted as a vital enabler or major barrier [19]. Put simply, where senior leaders are willing to engage, understand and respond to those from other sectors there is the opportunity for progress. Where this is not the case, however, fragmentation remains and opportunities to explore new flexibilities are not exploited [32]. Once again, the importance of trust between individuals and the necessity of forums and meetings in which constructive relationships between health and social care organisations can be fostered has been highlighted.

Alongside local factors there remain numerous other barriers that restrict integration. While pooling of funds is possible, the legislative framework makes this a complex and arduous task due to a requirement on individual organisations to safeguard their own financial position [30]. There are different regulations regarding Value Added Tax (a general tax on goods and services) for NHS organisations, independent providers and local authorities which can result in unaffordable tax bills being levied on new organisational partnerships [30]. Major problems with transferring staff between sectors due to pension differences also remain [30]. Similar challenges relate to siloed regulatory regimes and to STPs and ICSs not having the legislative status of ‘statutory bodies’. They do not have formal legal authority, and runs the risk of undermining transparency and accountability [30]. The discrepancies between health and social care present numerous difficulties. These include: the financial insecurity of social care providers and thereby sustainability of the market; enormous challenges concerning the recruitment and retention of social care staff; considerable differences in terms of pay and the status of the health and social care workforces; and, the sheer complexity of social care funding. National bodies have also been seen to have most interest in those areas making greatest progress to the practical and financial detriment of areas finding integration harder to achieve [29]. Finally, unprecedented cuts to local authorities and related public bodies has led to a decline in spending on social determinants of health with more deprived areas and populations being disproportionally affected [21].

The continued fragmentation between health and social care was perhaps most acutely demonstrated during the early stages COVID 19 pandemic. The National Audit Office [37] reported that responding to the emerging situation was “undoubtedly made harder because of historic and unaddressed differences and divisions between the two sectors” [p4]. It appeared that the focus of government was on ensuring that hospitals had sufficient capacity with insufficient attention paid to the risks of the virus spreading to residents and staff within care homes. For examples, there was an initial lack of requirement for all patients discharged from hospital to have a COVID test and an expectation that care homes must fill their capacity and admit patients with COVID 19. Testing was subsequently introduced on discharge due to numerous outbreaks within care homes. Similarly, whilst there were problems in relation to clarity of guidance and practical access to personal protective equipment in both sectors, social care providers raised concerns that the advice was tailored to health care settings and that they had even greater difficulty in obtaining sufficient supplies [38]. It is worth noting that the major discrepancies between health and social care relate to decisions taken at the national level. Within local areas, there were numerous examples of constructive collaboration with reports that the scale and urgency of the response resulted in the “breaking down of longstanding boundaries” between health and social care [39 p5].

## Discussion

So what might we take from the last few decades of England’s experience of integrated care? In one sense this is a success story. It is clear that integrated care matters. Under governments of different hues, although the terminology has changed, integrated care has remained a key area of policy focus and of significant local activity and commitment. For all of the well-rehearsed difficulties associated with integrating care, health and social care professionals know that they need to work together to deal with many of the pressing challenges that their organisations and communities face. While progress can be slow and incredibly frustrating, professionals, local services and policy makers all want to make integration work – and what was once something of a ‘bolt-on’ to traditional ways of working has now become part of the mainstream.

Despite these changes in emphasis and focus, the greatest progress has arguably been made when local areas have focused on specific tangible tasks or issues. To some extent this is not a new observation, with some of the authors noting this in relation to joint commissioning nearly a decade ago [33]. Where local aspirations for integrated care are broad and amorphous (e.g. ‘reduce health inequities’, ‘improve care’) there is greater room for this to be interpreted in multiple ways and this is less able to galvanize local action. Where specific locally-relevant aspirations are articulated that a range of partners are able to buy into or take ownership of, these have a greater chance of being achieved. Individuals and their organisations are more likely to put aside differences and be willing to take the risk of trusting each other when they can see tangible benefits. What this means is that integrated care initiatives need to be clear about what they are trying to achieve and for whom, design their approach with this in mind, and regularly measure their performance against these goals in order to be effective.

In other ways, however, the English experience is less of a success story. There is still too much of a tendency to see integration as somewhat of a prescription for all ills. As such there is a tendency to over-promise in terms of what can be delivered and this inevitably sets some initiatives up to fail. We need to have more realistic views of what can be achieved through integration and the amount of time that it takes to achieve this – particularly when setting up new initiatives (or even new organisations) from scratch. While we may see some early impacts around delayed transfers of care or emergency readmissions to hospitals, it might take longer to see significant impacts or to see broader changes (such as a reduction in health inequalities). If we expect too much of these integration arrangements, we are setting them up to fail. This can disengage staff and those using these services as they will lose trust in the promised made by those in more senior roles within partnerships.

One of the issues we have clearly seen is that a hyperactive policy context is not helpful to developing and sustaining joint activity. Over at least the last ten years, we have seen a layering of new policy on top of old policy and a number of pilots that have been established but not maintained for sufficiently long to have an influence on mainstream services. Just over a decade ago, Professor Kieran Walshe [34] described the continual reorganisation of the NHS as creating organisational ‘shanty towns’, where new entities were hastily constructed, knowing that they too would soon be swept away. The recent experience of integrated care again demonstrates that this kind of frenetic policy context is not helpful to creating and maintaining relationships or to building for the long-term. Rather than simply bolting on new policy reforms or new agencies we would argue we need to fundamentally change the underlying system if we are serious about making integrated care work.

Walter Leutz [35] famously set out his five laws of integration and his fourth point was that ‘you can’t integrate a square peg into a round hole’. This law is sometimes interpreted to mean that you cannot prescribe one approach to integration and all approaches need to be locally developed. The English experience demonstrates this to be true. But Leutz, in making this point, was also indicating that some systems are unable to be integrated because they simply do not fit together. We might argue that after more than two decades of significant effort to drive integration, any lack of further progress is unlikely to be simply due to a lack of will or effort. Instead, it is likely that there is a more fundamental issue at the core of this lack of widespread success. English health and social care services were simply not designed either as a system or with integration in mind. This issue has arguably become even clearer over the last decade as we have seen social care starved of funding. As a result, it remains the poor relation of the NHS. All too often policies badged as being about ‘integrated care’ default to health-related outcomes and to hospital care (which is the most powerful and best resourced part of the current system). Unless we see significant investment in social care and pay attention to the underlying funding of core services then it will remain difficult to drive integration much further and maintain progress on a long term basis.

## Conclusion

As 2020 came to an end, Integrated Care Systems were confirmed to be the principal vehicle through which greater collaboration is to be achieved in the decade to follow. A consultation document set out proposals that would address a number of the concerns outlined above [36]. This includes giving Integrated Care Systems a stronger footing in legislation, aligning priorities through a common ‘triple aim’ duty on all NHS organisations, and developing a ‘single’ health care funding pot with local freedoms. All worthy developments, but the document is undeniably still NHS-centric with local authorities and the voluntary sector getting scant mention other than being noted as important partners to the NHS. Even less considered is how people and communities will be able to influence the work of these powerful new bodies. Casting our minds back to the beginning of the decade, this presents a contrasting image to the shared vision launched by the National Collaboration. More people-centric in tone, it suggested greater equality between health and social care, the NHS and local authorities, and the statutory and voluntary sectors. Instead, the image portrayed is that Integrated Care Systems are at their heart a health care concern. If this proves to be the case, then much of the good work from the 2010’s will be lost. This would be a travesty, particularly in light of the acceleration in collaboration between health and social care that was experienced by many local areas by the end of the first year of the COVID 19 response. The pandemic has left local authorities and therefore social care in an even worse financial position and the NHS has accumulated huge waiting lists for planned care procedures. There is therefore a significant danger that these sectors may retreat to focus on their own pressures. One must hope that other countries can still learn from what went well and that, in time, England will regain its vision for a more holistic and equitable health and social care system.
